# Supplementary material for: Redirecting immune signaling with cytokine adaptors
Source: Nat Commun. 2025 Mar 11;16:2432. doi: 10.1038/s41467-025-57681-1 (PMC11897282; doi:10.1038/s41467-025-57681-1)
Supplement: Supplementary file 1 — Supplementary Information [file 41467_2025_57681_MOESM1_ESM.pdf]

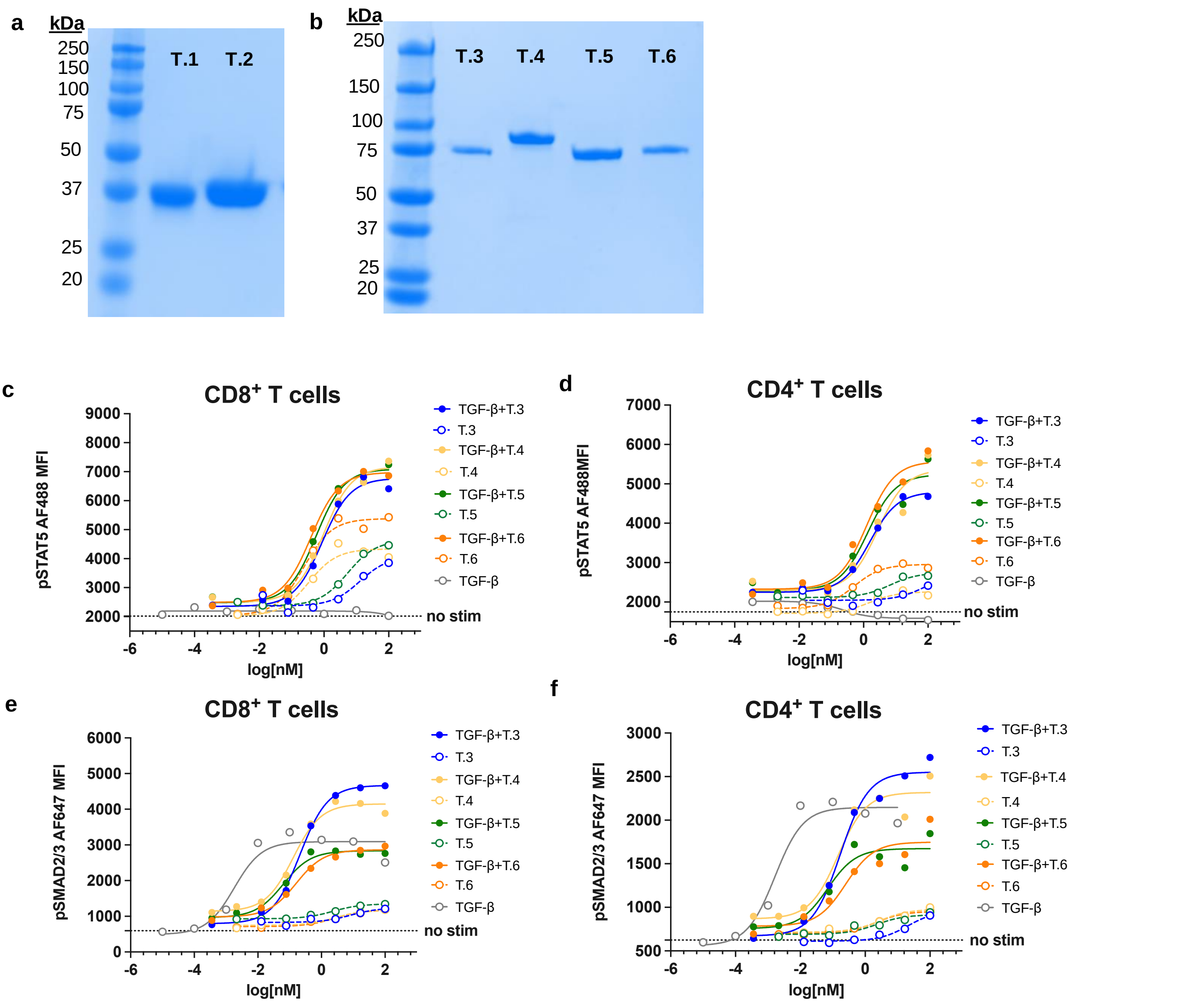

Supplementary Fig. 1. Expression and optimization of single-chain TGF-β→IL-2 adaptors.

(a) Coomassie-stained SDS-PAGE gel of adaptor T.1 and T.2. kDa = kilodalton. **(b)** Coomassie-stained SDS-PAGE gel of adaptors T.3. **(c–f)** Dose response curves for pSTAT5 and pSMAD2/pSMAD3 in CD4^{+} and CD8^{+} human T cells stimulated with adaptors T.3, T.4, T.5, and T.6 with or without equimolar TGF-β. Data represents mean fluorescence intensity at the indicated concentration (n=2).

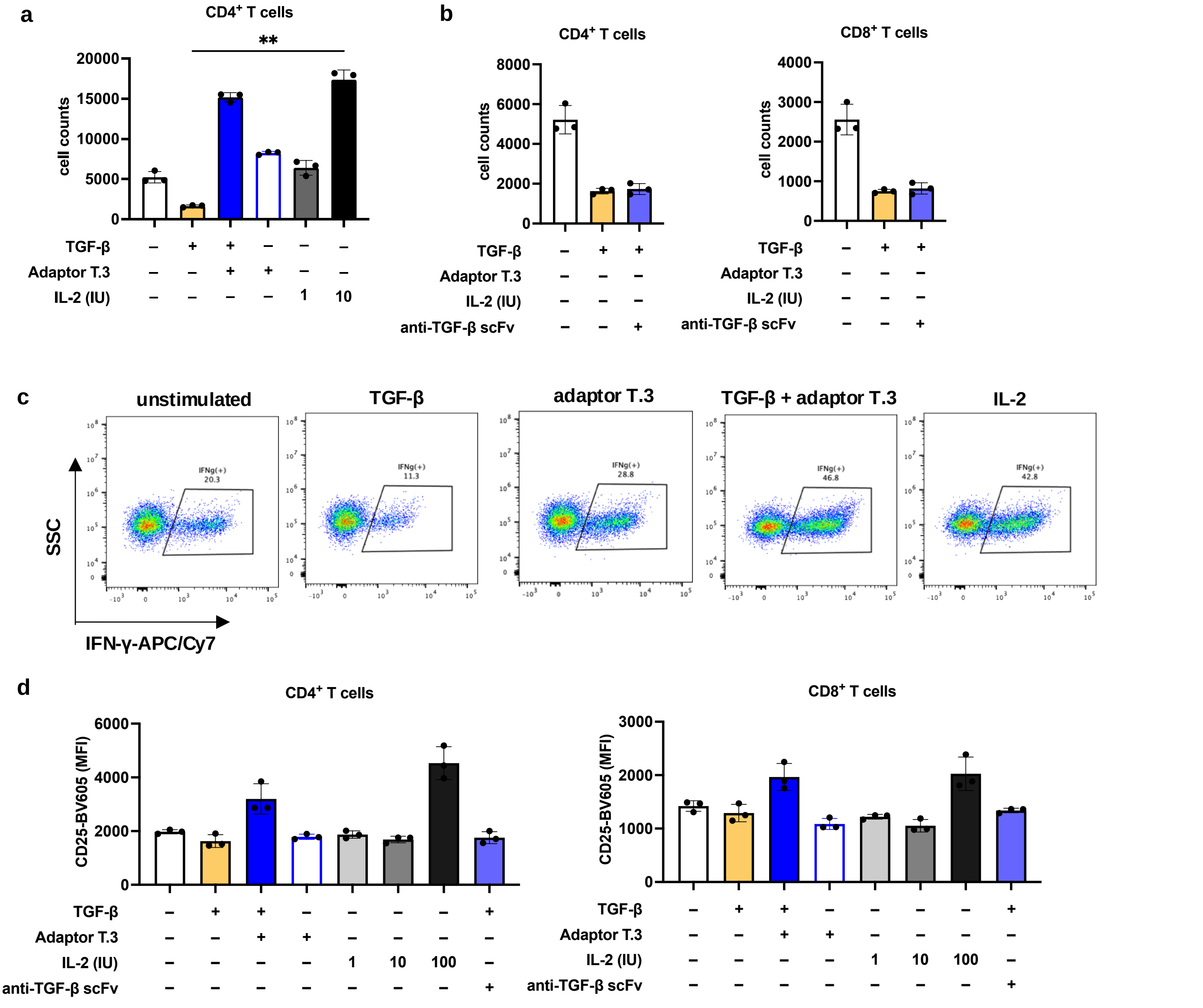

Supplementary Fig. 2.

(a) CD4+ T cell counts after culture for 6 days +/- 500ng/mL TGF-β +/- equimolar Adaptor T.3, or IL-2 (1 or 10 IU). N=3 replicates per condition. Bar graphs represent mean ± SD. **(b)** CD4+ or CD8+ T cell counts after 6 days +/- 500ng/mL TGF-β +/- equimolar anti-TGF-β scFv. n=3. Bar graphs represent mean ± SD. **(c)** Representative flow plots of IFN-γ staining in human CD8+ T cells stimulated with 500ng/mL TGF-β, adaptor T.3, TGF-β and adaptor T.3, or 100IU of IL-2. **(d)** CD25 mean fluorescence intensity measured on human CD4+ or CD8+ T cells cultured for 6 days +/- 500ng/mL TGF-β +/- equimolar Adaptor T.3, or IL-2 (1, 10, or 100IU). n=3. Bar graphs represent mean ± SD.

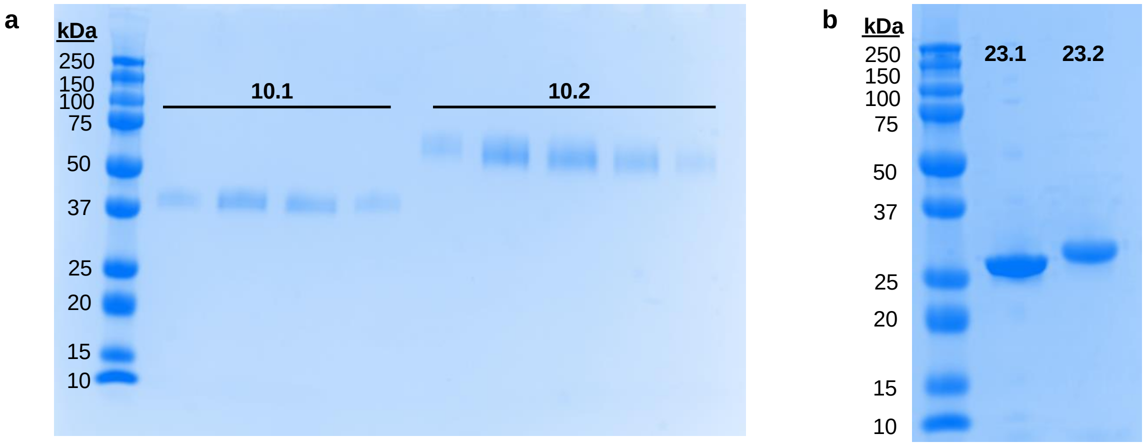

Supplementary Fig. 3. Expression of cytokine adaptors.

(a) Coomassie-stained SDS-PAGE gel of Adaptors 10.1 and 10.2 **(b)** Coomassie-stained SDS-PAGE gel of Adaptors 23.1 and 23.2.

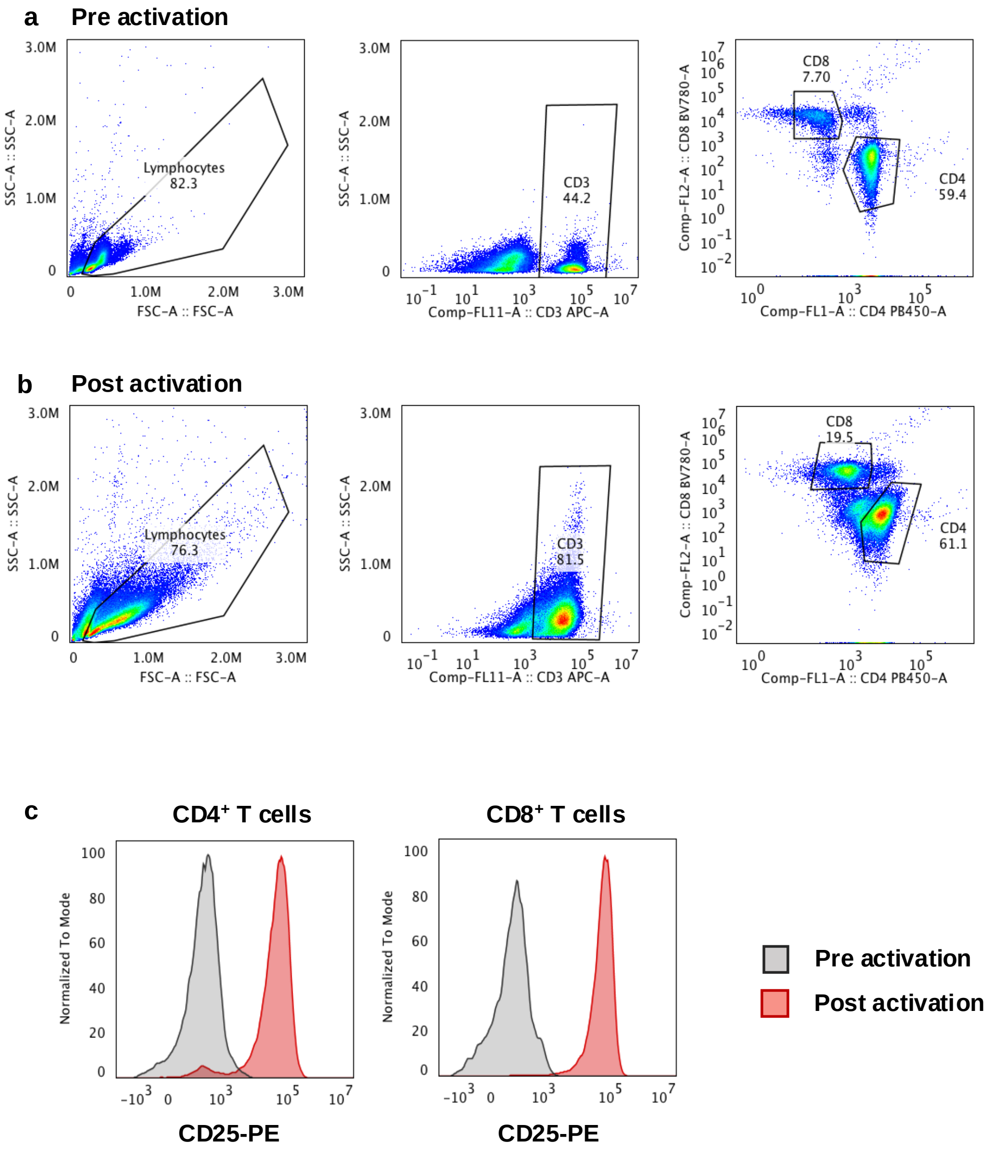

Supplementary Fig. 4. Gating of primary human T cells pre- and post- activation.

(a–b) Gating scheme on human PBMCs pre activation (a) or post activation for 48 hours with anti-CD3 coated plate, soluble anti-CD28, and soluble IL-2 (b). (**c**) Histogram of IL-2Rα (CD25) expression on $CD4^+$ and $CD8^+$ T cells pre and post activation.

Supplementary Table 1. Dose response characteristics of TGF-β→IL-2 adaptors in YT-1 cells, corresponding to Figure 1.

Ligand	EC_{50}	E_{max} (MFI)	E_{max} (%)
**IL-2**	1.2nM	36058	100%
**TGF-β + T.1 + T.2**	1.6nM	20031	57%

Supplementary Table 2. Dose response characteristics of TGF-β→IL-2 adaptors in primary T cells, corresponding to Figure 2.

Ligand	EC_{50}		E_{max} (MFI)		E_{max} (%)	
	$CD4^+$	$CD8^+$	$CD4^+$	$CD8^+$	$CD4^+$	$CD8^+$
**IL-2**	3.9pM	3.1pM	31473	24652	100%	100%
**TGF-β + T.1 + T.2**	6.1nM	7.0nM	21558	20380	61%	77%
**TGF-β + T.3**	810pM	900pM	28222	24232	87%	98%

Supplementary Table 3. Dose response characteristics of IL-10→IL-2 adaptors.

Ligand	EC_{50}	E_{max} (MFI)	E_{max} (normalized %)
**IL-2**	2.1nM	24133	100%
**IL-10 + 10.1 + 10.2**	1.9nM	18669	67%
**IL-10 + 10.3 + 10.4**	1.9nM	20980	72%

Supplementary Table 4. Dose response characteristics of IL-23→IL-10 adaptors.

Ligand	EC_{50}	E_{max} (MFI)	E_{max} (normalized %)
**IL-10**	250pM	925	100%
**MonoIL-10**	250pM	784	73%
**IL-23 + 23.1 + 23.2**	24pM	674	41%

Supplementary Table 5. Materials

**Antibodies**		
Anti-Stat3 (pY705) Alexa Fluor® 647, clone 4/P-STAT3	BD Biosciences	Cat#557815
Anti-Stat5 (pY694) Alexa Fluor® 488, clone 47/Stat5(pY694)	BD Biosciences	Cat#612598
Anti-Stat5 (pY694 Alexa Fluor® 647, clone 47/Stat5(pY694)	BD Biosciences	Cat#612599
Human TruStain FcX™ (Fc Receptor Blocking Solution)	BioLegend	Cat#422302
Pacific Blue™ anti-human CD4 Antibody, clone RPA-T4	BioLegend	Cat#300521
FITC anti-human CD4 Antibody, clone RPA-T4	BioLegend	Cat#300506
Brilliant Violet 785™ anti-human CD8 Antibody, clone SK1	BioLegend	Cat#344739
Brilliant Violet 605™ anti-human CD8a Antibody clone SK1	BioLegend	Cat#344742
PE anti-human CD8, clone SK1	BioLegend	Cat#980902
APC/Cyanine7 anti-human CD8 Antibody, clone SK1	BioLegend	Cat#344714
Brilliant Violet 605™ anti-mouse CD25 Antibody, clone PC61	BioLegend	Cat#102036
APC/Cyanine7 anti-human IFN-γ Antibody, clone 4S.B3	BioLegend	Cat#502530
Alexa Fluor® 647 Mouse Anti-Human IFN-γ, clone B27	BD Biosciences	Cat#557729
Ultra-LEAF™ Purified anti-human CD3 Antibody, clone OKT3 clone	BioLegend	Cat#317326
Ultra-LEAF™ Purified anti-human CD28 Antibody, clone 28.2	BioLegend	Cat#302934
Alexa Fluor® 647 Anti-Smad2 (pS465/pS467)/Smad3 (pS423/pS425), clone O72-670	BD Biosciences	Cat#562696
PE anti-human CD25 Antibody, clone BC96	BioLegend	Cat#302605
APC anti-human CD3 Antibody, clone SK7	BioLegend	Cat#344812
**Bacterial strains**		
Mix & Go Competent Cells – DH5α	Zymo Research	Cat#T3007
**Chemicals, peptides, proteins**		
Phosphate Buffered Saline (PBS)	Gibco	Cat#20012-050
Fetal Bovine Serum	Sigma	Cat#F4135-500
NotI-HF	NEB	Cat#R3189L
BamHI-HF	NEB	Cat#R3136L
NheI-HF	NEB	Cat#R3131L
1-Step™ Ultra TMB-ELISA Substrate Solution	Thermo Fisher	Cat#34028
16% paraformaldehyde	Fisher Scientific	Cat#Cat#50-980-487
Bovine Serum Albumin, Fraction V	Fisher	Cat#BP1605-100
beta-mercaptoethanol	Thermo Fisher	Cat#21985023
Propidium iodide	Thermo Fisher	Cat#P3566
RPMI 1640 Medium	Sigma	Cat#R8758-24X500ML
MEM non-essential amino acids	Gibco	Cat#11140050
Sodium pyruvate	Gibco	Cat#11360-070
1M HEPES	Gibco	Cat#15630-080
Penicillin-streptomycin	Gibco	Cat#15-140-163
Recombinant Human IL-23 Protein	R&D	Cat#1290-IL-010
Recombinant Human TGF-beta 1 Protein	R&D	Cat#240-B-002
**Commercial assays**		
ExpiFectamine 293 Transfection Kit	Thermo Fisher	Cat#A14525
ELISA MAX™ Deluxe Set Human IFN-γ	BioLegend	Cat#430104
Nunc MaxiSorp ELISA plates, uncoated	BioLegend	Cat#423501
Human IL-1 beta/IL-1F2 DuoSet ELISA	R&D	Cat#DY201-05
Human IL-6 DuoSet ELISA	R&D	Cat#DY206-05
Human TNF-alpha DuoSet ELISA	R&D	Cat#DY210-05
**Cell lines**		
Human: Expi293F	Thermo Fisher	Cat#A14528
Human: YT-1	Cellosaurus	CVCL_EJ05
Human: THP-1	ATCC	TIB-202
**Software and algorithms**		
FlowJo v10.5	Tree Star	RRID: SCR_008520
GraphPad Prism 9.3.0	GraphPad Software	RRID: SCR_002798
UCSF ChimeraX	Goddard et al., 2018	RRID: SCR_015872
AlphaFold2	Jumper et al., 2021	
ColabFold	Mirdita et al., 2022	
Microsoft PowerPoint	Version 16.92	
