# Supplementary material for: Redirecting immune signaling with cytokine adaptors
Source: Nat Commun. 2025 Mar 11;16:2432. doi: 10.1038/s41467-025-57681-1 (PMC11897282; doi:10.1038/s41467-025-57681-1)
Supplement: Supplementary file 2 — Reporting Summary [file 41467_2025_57681_MOESM2_ESM.pdf]

nature portfolio

Corresponding author(s): K Christopher Garcia

Last updated by author(s): Feb 26, 2025

Reporting Summary

Nature Portfolio wishes to improve the reproducibility of the work that we publish. This form provides structure for consistency and transparency in reporting. For further information on Nature Portfolio policies, see our Editorial Policies and the Editorial Policy Checklist.

Statistics

For all statistical analyses, confirm that the following items are present in the figure legend, table legend, main text, or Methods section.

n/a	Confirmed	
☐	☒	The exact sample size (*n*) for each experimental group/condition, given as a discrete number and unit of measurement
☐	☒	A statement on whether measurements were taken from distinct samples or whether the same sample was measured repeatedly
☐	☒	The statistical test(s) used AND whether they are one- or two-sided<br>*Only common tests should be described solely by name; describe more complex techniques in the Methods section.*
☒	☐	A description of all covariates tested
☐	☒	A description of any assumptions or corrections, such as tests of normality and adjustment for multiple comparisons
☐	☒	A full description of the statistical parameters including central tendency (e.g. means) or other basic estimates (e.g. regression coefficient) AND variation (e.g. standard deviation) or associated estimates of uncertainty (e.g. confidence intervals)
☐	☒	For null hypothesis testing, the test statistic (e.g. *F*, *t*, *r*) with confidence intervals, effect sizes, degrees of freedom and *P* value noted<br>*Give P values as exact values whenever suitable.*
☒	☐	For Bayesian analysis, information on the choice of priors and Markov chain Monte Carlo settings
☒	☐	For hierarchical and complex designs, identification of the appropriate level for tests and full reporting of outcomes
☒	☐	Estimates of effect sizes (e.g. Cohen's *d*, Pearson's *r*), indicating how they were calculated

Our web collection on statistics for biologists contains articles on many of the points above.

Software and code

Policy information about availability of computer code

Data collection	ELISA assays were read on a SepctraMax Paradigm plate reader (Molecular Devices) using SoftMax Pro v7.1.<br>Signaling assays were read on a CytoFlex flow cytometer (Beckman Coulter) using CytExpert software (Beckman Coulter).
Data analysis	For cell-based assays including signaling data, proliferation, immunohistochemistry, and ELISAs, analyses were performed with GraphPad Prism v 9.3.0, RRID: SCR_002798.<br>For structure-related figures, analyses were conducted with ChimeraX v1.4 (Goddard et al. 2018).

For manuscripts utilizing custom algorithms or software that are central to the research but not yet described in published literature, software must be made available to editors and reviewers. We strongly encourage code deposition in a community repository (e.g. GitHub). See the Nature Portfolio guidelines for submitting code & software for further information.

Data

Policy information about availability of data

All manuscripts must include a data availability statement. This statement should provide the following information, where applicable:

- Accession codes, unique identifiers, or web links for publicly available datasets
- A description of any restrictions on data availability
- For clinical datasets or third party data, please ensure that the statement adheres to our policy

Data are available in the Source Data file.

Research involving human participants, their data, or biological material

Policy information about studies with human participants or human data. See also policy information about sex, gender (identity/presentation), and sexual orientation and race, ethnicity and racism.

Reporting on sex and gender	Information about sex was not collected for the cell types used in our in vitro assays, as we and others have previously not found an association between sex and cytokine signaling in vitro. Larger studies powered for this purpose will be necessary to determine whether there is an affect of sex on these therapies.
Reporting on race, ethnicity, or other socially relevant groupings	Information about race or ethnicity was not collected for the cell types used in our in vitro assays. Larger studies powered for this purpose will be necessary to determine whether there is an affect of race/ethnicity on these therapies.
Population characteristics	PBMCs from healthy donors were obtained from the Stanford Blood Center.
Recruitment	*Describe how participants were recruited. Outline any potential self-selection bias or other biases that may be present and how these are likely to impact results.*
Ethics oversight	*Identify the organization(s) that approved the study protocol.*

Note that full information on the approval of the study protocol must also be provided in the manuscript.

Field-specific reporting

Please select the one below that is the best fit for your research. If you are not sure, read the appropriate sections before making your selection.

[x] Life sciences [ ] Behavioural & social sciences [ ] Ecological, evolutionary & environmental sciences

For a reference copy of the document with all sections, see nature.com/documents/nr-reporting-summary-flat.pdf

Life sciences study design

All studies must disclose on these points even when the disclosure is negative.

Sample size	For signaling assays, sample sizes were determined based on previous experience with similar experiments (Saxton et al. 2021; Glassman et al. 2021). At least 10,000 cells were analyzed per condition by flow cytometry.
Data exclusions	No data were excluded.
Replication	All attempts at replication were successful in at least three biological donors or independent experiments.
Randomization	Human PBMCs were obtained from multiple donors at random. The experiments were not randomized. Investigators were not blinded to allocation during experiments and outcome assessment.
Blinding	Blinding of investigators was not performed or necessary due to readouts of signaling assays and ELISA assays not being subject to investigator bias.

Reporting for specific materials, systems and methods

We require information from authors about some types of materials, experimental systems and methods used in many studies. Here, indicate whether each material, system or method listed is relevant to your study. If you are not sure if a list item applies to your research, read the appropriate section before selecting a response.

Materials & experimental systems

n/a	Involved in the study	
[ ]	[x]	Antibodies
[ ]	[x]	Eukaryotic cell lines
[x]	[ ]	Palaeontology and archaeology
[x]	[ ]	Animals and other organisms
[x]	[ ]	Clinical data
[x]	[ ]	Dual use research of concern
[x]	[ ]	Plants

Methods

n/a	Involved in the study	
[x]	[ ]	ChIP-seq
[ ]	[x]	Flow cytometry
[x]	[ ]	MRI-based neuroimaging

Antibodies

Antibodies used	Anti-Stat3 (pY705) Alexa Fluor® 647, clone 4/P-STAT3

BD Biosciences
Cat#557815
Anti-Stat5 (pY694) Alexa Fluor® 488, clone 47/Stat5(pY694)
BD Biosciences
Cat#612598
Anti-Stat5 (pY694 Alexa Fluor® 647, clone 47/Stat5(pY694)
BD Biosciences
Cat#612599
Human TruStain FcX™ (Fc Receptor Blocking Solution)
BioLegend
Cat#422302
Pacific Blue™ anti-human CD4 Antibody, clone RPA-T4
BioLegend
Cat#300521
FITC anti-human CD4 Antibody, clone RPA-T4
BioLegend
Cat#300506
Brilliant Violet 785™ anti-human CD8 Antibody, clone SK1
BioLegend
Cat#344739
Brilliant Violet 605™ anti-human CD8a Antibody clone SK1
BioLegend
Cat#344742
PE anti-human CD8, clone SK1
BioLegend
Cat#980902
APC/Cyanine7 anti-human CD8 Antibody, clone SK1
BioLegend
Cat#344714
Brilliant Violet 605™ anti-mouse CD25 Antibody, clone PC61
BioLegend
Cat#102036
APC/Cyanine7 anti-human IFN-γ Antibody, clone 4S.B3
BioLegend
Cat#502530
Alexa Fluor® 647 Mouse Anti-Human IFN-γ, clone B27
BD Biosciences
Cat#557729
Ultra-LEAF™ Purified anti-human CD3 Antibody, clone OKT3 clone
BioLegend
Cat#317326
Ultra-LEAF™ Purified anti-human CD28 Antibody, clone 28.2
BioLegend
Cat#302934
Alexa Fluor® 647 Anti-Smad2 (pS465/pS467)/Smad3 (pS423/pS425), clone O72-670
BD Biosciences
Cat#562696
PE anti-human CD25 Antibody, clone BC96
BioLegend
Cat#302605
APC anti-human CD3 Antibody, clone SK7
BioLegend
Cat#344812

Validation

All antibodies were validated by the vendors. Further validation details and references for each antibodies can be found at the BioLegend webpage (https://www.biolegend.com/de-at/reproducibility/committed-to-functional-quality) or BD Biosciences webpage (https://www.bdbiosciences.com/en-us/products/reagents/flow-cytometry-reagents/research-reagents/quality-and-reproducibility) as well as the page for each antibody as identified by catalog number.

Eukaryotic cell lines

Policy information about cell lines and Sex and Gender in Research

Cell line source(s)

Human: Expi293F Thermo Fisher Cat#A14528
Human: YT-1 Cellosaurus CVCL_EJ05
Human: THP-1 ATCC TIB-202

Authentication

Expi293F cells, YT-1 cells, and THP-1 cells were guaranteed by the suppliers and no additional authentication was performed

	by authors of this study.
Mycoplasma contamination	Cell lines were tested for Mycoplasma contamination at least once per year using MycoAlert (R) Mycoplasma detection kit (Lonza)
Commonly misidentified lines (See ICLAC register)	None of the cell lines used in this study are commonly misidentified.

Plants

Seed stocks	*Report on the source of all seed stocks or other plant material used. If applicable, state the seed stock centre and catalogue number. If plant specimens were collected from the field, describe the collection location, date and sampling procedures.*
Novel plant genotypes	*Describe the methods by which all novel plant genotypes were produced. This includes those generated by transgenic approaches, gene editing, chemical/radiation-based mutagenesis and hybridization. For transgenic lines, describe the transformation method, the number of independent lines analyzed and the generation upon which experiments were performed. For gene-edited lines, describe the editor used, the endogenous sequence targeted for editing, the targeting guide RNA sequence (if applicable) and how the editor was applied.*
Authentication	*Describe any authentication procedures for each seed stock used or novel genotype generated. Describe any experiments used to assess the effect of a mutation and, where applicable, how potential secondary effects (e.g. second site T-DNA insertions, mosiacism, off-target gene editing) were examined.*

Flow Cytometry

Plots

Confirm that:

- [x] The axis labels state the marker and fluorochrome used (e.g. CD4-FITC).
- [x] The axis scales are clearly visible. Include numbers along axes only for bottom left plot of group (a 'group' is an analysis of identical markers).
- [x] All plots are contour plots with outliers or pseudocolor plots.
- [x] A numerical value for number of cells or percentage (with statistics) is provided.

Methodology

Sample preparation	For dose-response signaling assays in YT-1 cells and THP-1 cells, cells were plated in a 96 well plate at 200,000 cells per well in RPMI. For signaling assays in activated human T cells, human peripheral blood mononuclear cells (PBMCs) were isolated from samples from the Stanford Blood Center and cryopreserved. Cytokines or cytokine adaptors were prepared at indicated concentrations and added to cells for 20 min at 37°C. Cells were fixed with 1.5% paraformaldehyde for 10 min at room temperature, then permeabilized with 100% methanol for 30 min at -20°C. Intracellular STAT activation was assayed by staining with Alexa Fluor® 488 or 647 conjugated anti-STAT5 (pY694) or anti-STAT3 (pY705) (BD) for 1 hour at room temperature. Mean fluorescence intensities were measured using a CytoFlex flow cytometer (Beckman Coulter) and analyzed in Prism v9.3.0 (GraphPad).
Instrument	CytoFlex (Beckman Coulter)
Software	CytExpert (Beckman Coulter)
Cell population abundance	200,000 cells were stimulated per condition and at least 10,000 cells were analyzed per condition.
Gating strategy	Figures exemplifying the gating strategy are provided in the Extended Data. For live cell counts, cells were gated on SSC-H and FSC-H, followed by gating on the propidium iodide negative populaion (live cells). Cells were further gated on CD3 and/or CD4, CD8. For intracellular cytokine staining, cells were gated on SSC-H and FSC-H, followed by gating on CD4, CD8, IFNg, or TNFa.

- [x] Tick this box to confirm that a figure exemplifying the gating strategy is provided in the Supplementary Information.
