# Supplementary material for: Redirecting immune signaling with cytokine adaptors
Source: Nat Commun. 2025 Mar 11;16:2432. doi: 10.1038/s41467-025-57681-1 (PMC11897282; doi:10.1038/s41467-025-57681-1)
Supplement: Supplementary file 4 — Source Data [file 41467_2025_57681_MOESM4_ESM.zip › SourceData/SourceData2.pdf]

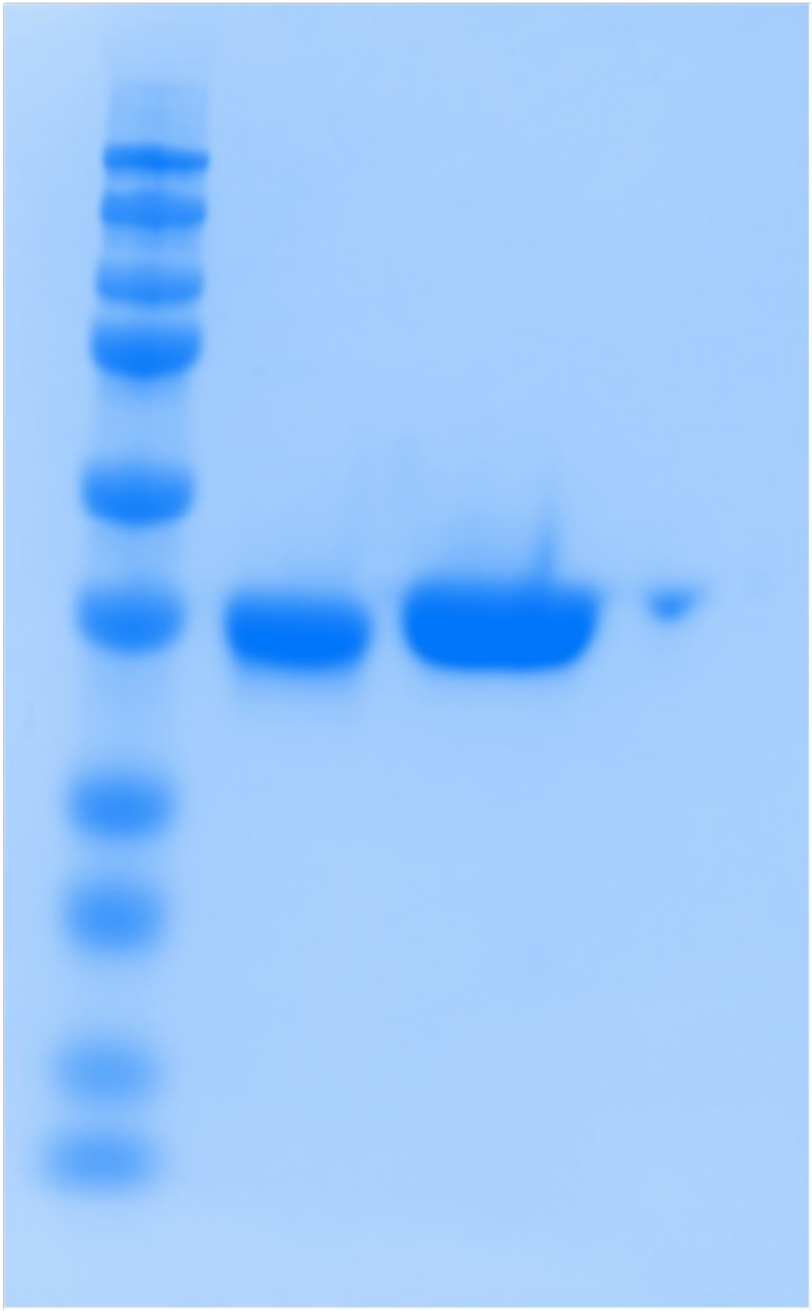

Gel for Supplementary Fig. 1a

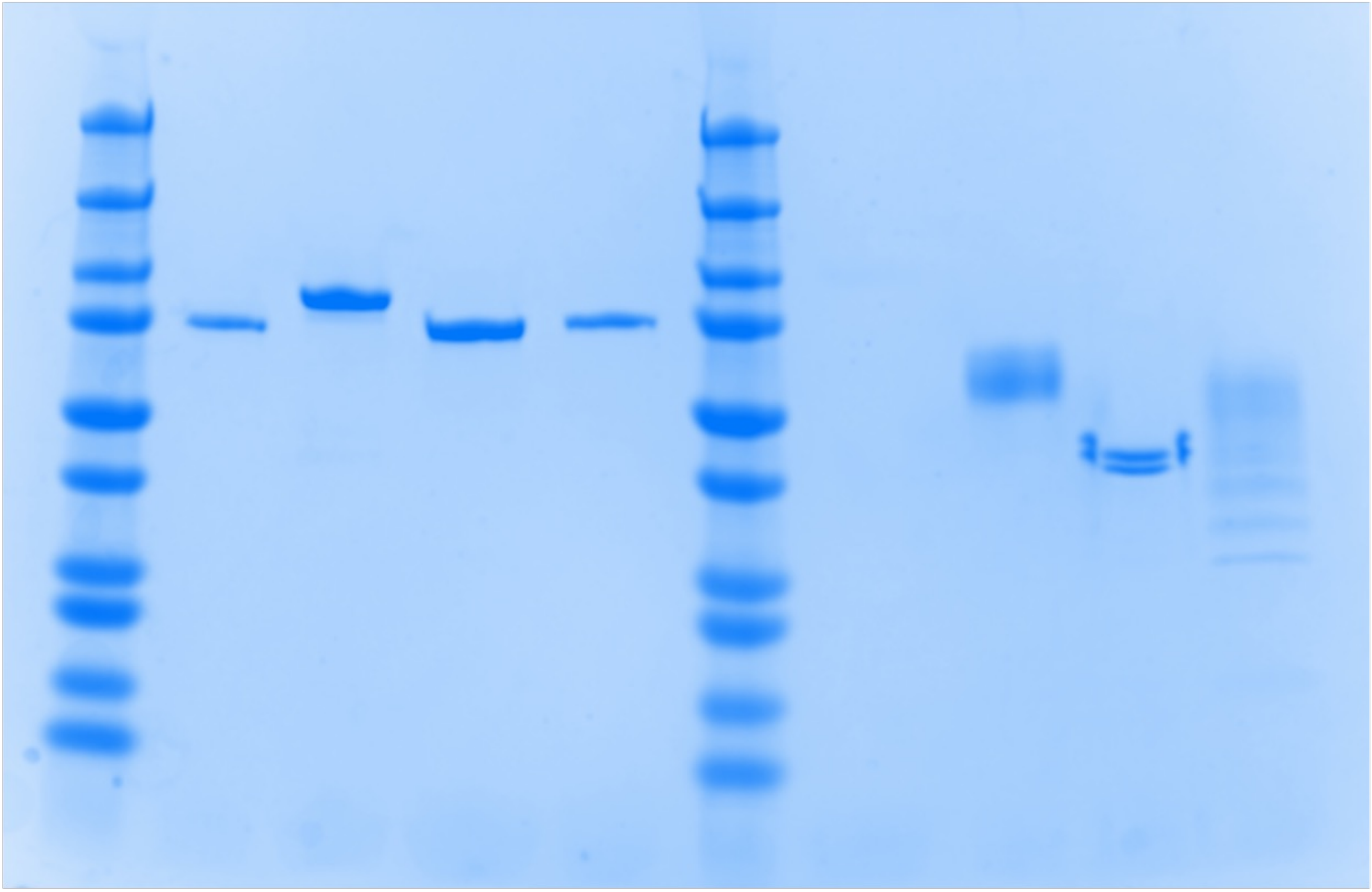

Gel for Supplementary Fig. 1b

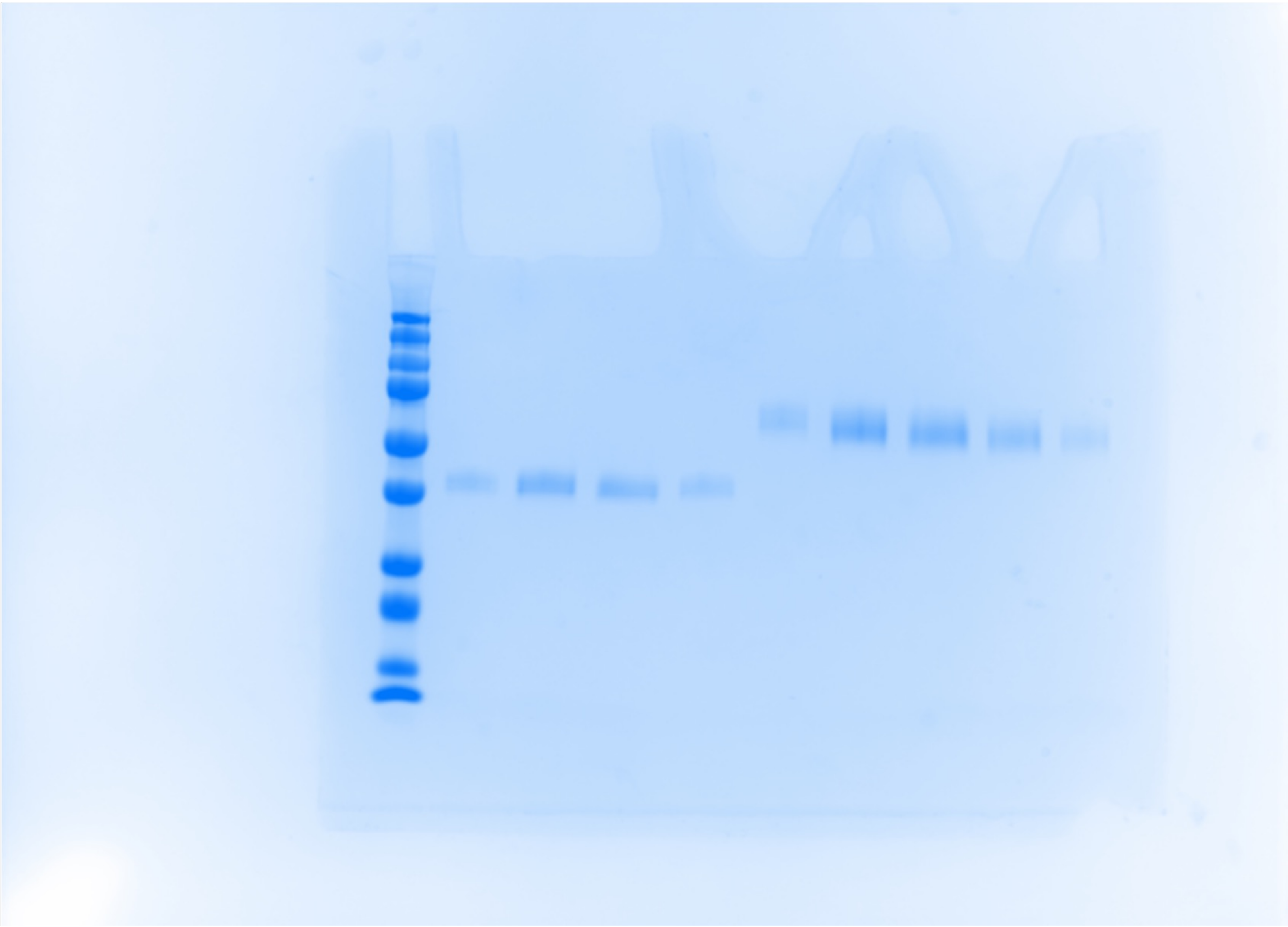

Gel for Supplementary Fig. 4a

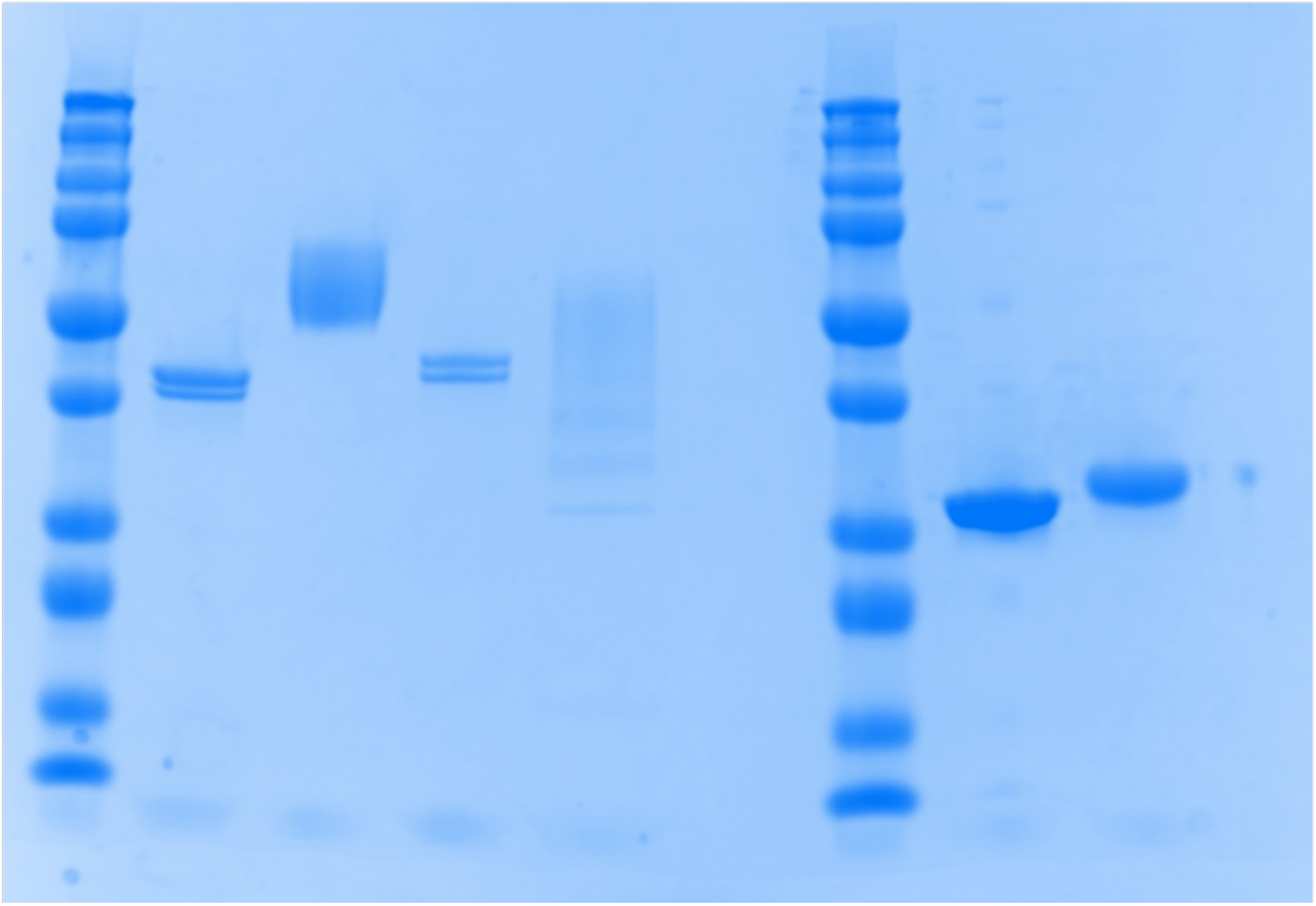

Gel for Supplementary Fig. 4b
